# Supplementary figures and images for: Mendelian randomization analyses clarify the effects of height on cardiovascular diseases
Source: PLoS One. 2024 Jul 3;19(7):e0298786. doi: 10.1371/journal.pone.0298786 (PMC11221663; doi:10.1371/journal.pone.0298786)

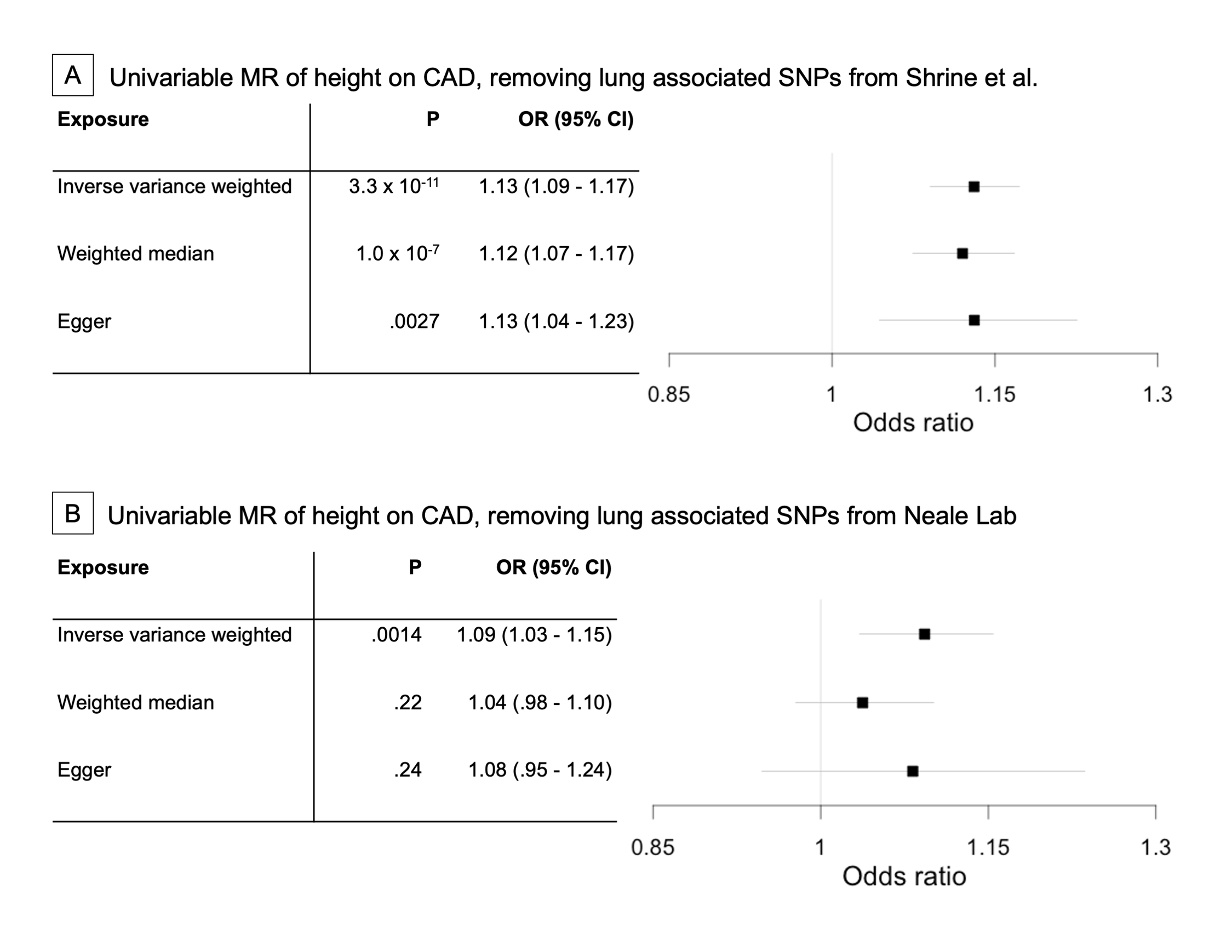

Supplement: S1 Fig — Univariable effect of 1-SD decrease in height on CAD risk after removing variants from set of height instruments that are nominally associated (p <0.05) with lung function from GWAS conducted by: (a) Shrine et al. (N SNPs = 1,112) and (b) Neale Lab (N SNPs = 747). (TIF) [file pone.0298786.s002.tif]
