# Supplementary material for: Mendelian randomization analyses clarify the effects of height on cardiovascular diseases
Source: PLoS One. 2024 Jul 3;19(7):e0298786. doi: 10.1371/journal.pone.0298786 (PMC11221663; doi:10.1371/journal.pone.0298786)
Supplement: Attachment — Submitted filename: HuiEA_Height_CVD_MR_Rebuttal_DH_v2.docx [file pone.0298786.s024.docx]

**We sincerely thank the reviewers and editors for their careful reading of the manuscript and their criticisms and suggestions. We have taken their feedback to heart in this revision, which we think has substantially improved this revised manuscript. Reviewer’s and Editor’s comments are in *italics*, and our specific responses are below in bold.**

**## Editorial Comments:**

*Journal Requirements:*

*When submitting your revision, we need you to address these additional requirements.*

*1. Please ensure that your manuscript meets PLOS ONE's style requirements, including those for file naming. The PLOS ONE style templates can be found at*
*https://journals.plos.org/plosone/s/file?id=wjVg/PLOSOne_formatting_sample_main_body.pdf and*
*https://journals.plos.org/plosone/s/file?id=ba62/PLOSOne_formatting_sample_title_authors_affiliations.pdf*

**Author Response: We have formatted this accordingly.**

*2. We note that the grant information you provided in the ‘Funding Information’ and ‘Financial Disclosure’ sections do not match.*

*When you resubmit, please ensure Tthat you provide the correct grant numbers for the awards you received for your study in the ‘Funding Information’ section.*

**Author Response: We have confirmed the funding acknowledgements and numbers listed on the online form submission.**

*3. Thank you for stating the following in the Acknowledgments Section of your manuscript:*

*This research is based on data from the Million Veteran Program, Office of Research and Development, Veterans Health Administration, and was supported by the Veterans Administration (VA) Cooperative Studies Program (CSP) award #G002. B.F.V. acknowledges support from the National Institutes of Health (DK126194 and DK101478) and Linda Pechenik Montague Investigator Award. C.S.T acknowledges support from the National Institutes of Health (HL156052). S.M.D. is supported by the U.S. Department of Veterans Affairs award IK2-CX001780. This publication does not represent the views of the Department of Veterans Affairs or the United States Government. The funders had no role in study design, data collection and analysis, decision to publish, or preparation of the manuscript.*

*We note that you have provided funding information that is not currently declared in your Funding Statement. However, funding information should not appear in the Acknowledgments section or other areas of your manuscript. We will only publish funding information present in the Funding Statement section of the online submission form.*
*Please remove any funding-related text from the manuscript and let us know how you would like to update your Funding Statement. Currently, your Funding Statement reads as follows:*

*NO - The funders had no role in study design, data collection and analysis, decision to publish, or preparation of the manuscript.*

*Please include your amended statements within your cover letter; we will change the online submission form on your behalf.*

**Author Response: We have removed the funding declarations from the main text. The Funding Statement as currently stated is acceptable and required no editing.**

*4. Thank you for stating the following in the Competing Interests section:*

*D.H., E.S., K.L., T.L.A., C.S.T., and B.F.V. have no conflicting interest to report. S.M.D receives research support from RenalytixAI and personal consulting fees from Calico Labs, outside the scope of the current research.*

*Please confirm that this does not alter your adherence to all PLOS ONE policies on sharing data and materials, by including the following statement: "This does not alter our adherence to PLOS ONE policies on sharing data and materials.” (as detailed online in our guide for authors http://journals.plos.org/plosone/s/competing-interests). If there are restrictions on sharing of data and/or materials, please state these. Please note that we cannot proceed with consideration of your article until this information has been declared.*

*Please include your updated Competing Interests statement in your cover letter; we will change the online submission form on your behalf.*

**Author Response: There are no data sharing restrictions and we have added that statement to the text.**

*5. We note that you have stated that you will provide repository information for your data at acceptance. Should your manuscript be accepted for publication, we will hold it until you provide the relevant accession numbers or DOIs necessary to access your data. If you wish to make changes to your Data Availability statement, please describe these changes in your cover letter and we will update your Data Availability statement to reflect the information you provide.*

**Author response: This referred to availability of code used for some parts of the analysis. The github link is public, active, and populated with code that we intend to share.**

*6. Please include captions for your Supporting Information files at the end of your manuscript, and update any in-text citations to match accordingly. Please see our Supporting Information guidelines for more information: http://journals.plos.org/plosone/s/supporting-information.*

**Author Response: We have updated the Supporting Info file listing at the end of the text, updated in-text citations to match to adhere to formatting guidelines.**

**## Reviewer Comments**

*Review Comments to the Author*

***Reviewer #1:***

*This is an interesting study on the effects of height on CAD and cardiovascular outcomes. The results confirm the causal effect of height on CAD, but this effect is significantly attenuated after conditioning on 12 epidemiological risk factors for CAD. The mediating role of lung function (FEV1 and FVC) is also explored in MVMR and univariate MR of lung function on CAD, and the null results contradict previous evidence. The results shed light on the association between height and CAD, and complement previous studies on the topic, both MR and epidemiological. Overall, the paper is well written and the methods are robust. My main concern is the overlap between GWAS populations tested in the two-sample MR setting of this study, which can lead to weak instrument or winner’s curse. This can be addressed using new MR methods that adjust IVW estimates taking into consideration sample overlap.*

**Author Response: We thank the reviewer for their positive comments.**

*Major comments:*

*7. As mentioned above, I am concerned about overlap of samples in the exposure and outcome GWAS. In the discussion , the authors mention the overlap of samples among GWAS used in the two-sample MR, but could they should further comment on the impact of this on the MR findings. New methods are available and can be applied in case of sample overlap between GWAS used in a two sample MR context to attenuate bias ( see Mounier & Kutalik, 2023; PMID: 37036286).*

**Author Response: It is possible to estimate the expected bias due to sample overlap over potential models between exposure and outcome (PMID: 27625185). Using this tool and parameters for our data (R2 = 0.264, n_exposure = 693529, n_outcome=547261, n_ivs = 2041, effect estimate=-0.1198), we expect essentially little if any bias (for perfect overlap, less than .1% bias) even for very large sample overlaps.**

*8. In the same direction, in table S1, it is important to provide additional descriptives of the cohorts, for instance, mentioning if UKBB or other biobanks were part of the GWAS meta-analyses, and the N of samples of these biobanks participating in each GWAS meta-analysis- this could help the reader appreciate better the extend of the overlap. Also, given the importance of adjustment for variables such as height in the various GWAS of epidemiological risk factors, please add a column in Table S1 indicating for which covariates the GWAS phenotype was adjusted. Please discuss on the implications of the different covariates used in the studies ( see Hartwig, 2021; PMID: 33619569).*

**Author Response: The UK Biobank individuals were the largely only source of sample overlaps. We have added the number of individuals per each GWAS to S1 Table. Covariates used in each respective GWAS have also been added to S1 Table.**

*9. Results: is the reported F-statistic a mean F-statistic or a cumulative one? I would assume a much larger cumulative F-statistic.*

**Author Response: These are mean statistics. Cragg-Donald F-statistics are provided in Table S4.**

*10. The authors claim that there is a 12% increase in risk of CAD in the univariate MR analysis, but the actual IVW OR is 1.13, shouldn't that be 13% then?*

**Author Response: Thanks to the reviewer for catching this rounding bug. The estimate is 11.7% with an OR of 1.12. We have modified the main text and Figure 2 with this correction.**

*11. It would be helpful to provide estimates of pleiotropy for the MR instruments- such as the MR-Egger intercept p-value, Cohran Q heterogeneity estimates, for all the univariate MR analyses- and comment on these.*

**Author Response: MR-Egger Intercept p-values are included in S5, S13, S14, S19, and S20 Tables. We also use the random effects model in IVW, anticipating that Cochran’s Q statistic is frequently highly significant.**

*12. Also, given the increased number of SNP-instruments notably for height, additional sensitivity analyses such as the MR-PRESSO and the leave one out analysis could identify outlier SNPs and remove them. The same applies to MR studies using as exposure FEV1 or FVC.*

**Author Response: We performed MR-PRESSO and included results for outlier filtering in S6 Table. Overall, there was quantitatively and qualitative no difference between inclusion or exclusion of outliers relative to univariable MR results.**

*13. Methods: The clump R2 of 0.05 used to define independent SNPs for height and FVC-FEV1 could be liberal- the default clumping R2 in the TwoSampleMR package is 0.001. The authors should consider a more stringent clumping R2.*

**Author Response: We agree with the review Previous work utilized this much more liberal LD threshold; and thus in order to make sure we could reproduce and compare to their results, we opted to employ that same criteria.**

**To the reviewer specifically -- if we apply the threshold and filter the instrument further (r2 = 0.001), we retain 1,193 Height SNPs that are also present in both the Height and CAD study. Quantitatively, the estimates are similar after filtering, so prefer to retain our previous results given our desire to ultimately compare against:**

| Method | Beta | P |
| --- | --- | --- |
| IVW | -0.120767994 | 6.46E-11 |
| Weighted median | -0.135091869 | 8.66E-11 |
| Egger | -0.101970937 | 0.015606693 |

*Minor comments:*

*14. Introduction, last paragraph: there is the “factors” word missing at the end of a sentence: “12 established risk”. Also, in the same line, an “on” is missing after the “effects of height”.*

**Author Response: Yes, thanks for catching. We have edited accordingly to make this clear.**

*15. The term legacy datasets is used but it infers that some datasets might be obsolete. I suggest replacing it with “publicly available” datasets.*

**Author Response: Agreed and edited.**

*16. Methods, paragraph : “Generation of additional instrument sets for FEV1 and FVC”: “Alleles and effect sizes were harmonized to the height-increasing effect allele”. Do the authors mean FEV1 or FVC-increasing alleles?*

**Author Response: Agreed and edited.**

*17a. Paragraph “multivariate analysis”: last sentence, there is a typo: attenuation of the effect of height of CAD – it should be on CAD instead.*

**Author Response: Agreed and edited.**

*17b. Lung function measures are not genetically causally associated with CAD susceptibility: this does not read well. Genetically determined lung function is not causally associated with CAD*

**Author Response: Agreed and edited.**

*18. Discussion: The authors state that ”Our findings refute the notion that height, height-related nutritional status, or socioeconomic status meaningfully contributes to CAD risk (4)”. Based on the findings of the present study, one can argue that height does not have an important contribution to CAD risk, but nutritional status and socioeconomic status could actually play a role (see Figure 2) based on the results of the MVMR- but independently from height.*

**Author Response: We appreciate the reviewer’s comment; we feel this is a bit strong given that this evidence would derive from a relatively ‘weak’ instrument for SES, as I think the reviewer is taken by proxy from educational attainment. We have revised this point in discussion.**

*19. In Figure 2. the direction of effect of educational attainment is towards an increasing risk of CAD. Have the authors aligned SNPs for educational attainment to a decreasing effect for this exposure? This should be made clear, since miss-interpretation can occur.*

**Author Response: Yes, in figures we have listed the “Effect direction” of the exposure that is given to return a positive odds ratio on the the output, i.e., lower Ed attainment is associated here with elevated CAD risk.**

*19b.* *Please consider filling out the MR-STROBE ckecklist.*

**Author Response: We have prepared an MR-STROBE checklist to include as an S1 File in our revision.**

**Reviewer #2:**

*Hui et al. used a MR approach to evaluate the effect of height on coronary artery disease and other cardiovascular diseases. The authors use both univariable models and multivariable models adjusting for known CAD risk factors. They also consider a possible mediation of the effect of height on CAD by lung function phenotypes and reach the conclusion that there are no substantial mediating effect. This contrasts with previous reports that suggested that lung function was the major mediator of the height--CAD relationship. Overall, the report is clear and well written and the methodology is robust although I have some concerns listed below.*

**Author Response: We thank the reviewer for their positive sentiments and a careful reading of the work.**

*Major comments*

*20. The set of variants used as an instrument for height explains a large proportion of the heritability, which is desirable to attenuate the risk of weak instrument bias. However, the bias due to violations of the exclusion restriction assumption (i.e. IV independant from outcome given exposure) are not discussed. In this study, ~2000 variants spanning a large portion of the genome are used and certainly have direct effects on CAD either through unmeasured pathways or through LD with CAD risk loci biasing the MR estimates. As a sensitivity analysis, instruments (robustly) associated with height and with few other anticipated phenotypic effects should be used. Ideally, the variants included in such an analysis could be argued, biologically, to have direct effects on height.*

**Author Response: We certainly agree with the reviewer’s intuition here. However, in practice, this turns out to be fraught. If we filtered any SNP with nominal P < 0.05 association with any of the 12 GWAS used in our MVMR experiments, we only have 10 height SNPs remaining, which is woefully inappropriate to use for this experiment. Thus, we think the analytical framework of MVMR which directly includes the effects of potential mediating traits provides an appropriate approach to address this.**

*21. In the multivariable models, body mass index is included. In my view, since BMI is calculated using height (and weight), this complexifies the interpretation of the results. For instance, an attenuation would be expected as part of the heigh effect may be attributed to the BMI coefficient. In an MR setting based on individual level data, this would lead to multicolinearity issues which is numerically avoided in the summary statistics setting. However, here, the height coefficient can't be interpreted independently from the BMI coefficient.*

**Author Response: We certainly agree with the reviewer but are unsure how to fully reconcile this issue. If we exclude BMI entirely from the MVMR experiment, we certainly still see attenuation of Height on CAD (beta = -0.0648), but we know for sure that obesity is a risk factor for CAD so are excluding the effect of that on CAD. We have added a point in the limitations section.**

*22. Did the authors consider formal mediation analysis (e.g. Burgess IJE 2015) which seem appropriate to answer the question of mediation of height -> lung function -> cardiovascular disease.*

**Author Response: Given the accumulation of evidence here and the effort to clarify existing results, we did not pursue this line of inquiry. The evidence of any effect here seems, at best, slightly at most (null at the worst) and as such did not think this adds much value to the given presentation.**

*Minor comments*

*23. Given the large number of variants included as a genetic instrument of heigh and the fact that it's impossible to account for all possible biasing paths using pre-selected risk factors, the authors could have used unsupervised MR models that flexibly accounts for genetic effect heterogeneity. For example, the MR-Clust algorithm (Foley et al. Bioinformatics 2020) uses a mixture model to estimate MR effects that can vary. A post-hoc analysis of the inferred clusters of genetic instruments using summary statistics for known risk factors could then inform the main modulatory risk factors. I know that this approach is quite different from the current report, but perhaps it could be discussed in the future work or limitations section.*

**Author Response: We are certainly aware of and intrigued by this and other types of within-instrument clustering methods (e.g., the reviewer is undoubtably aware of NAvMix). As instruments get substantially larger (for traits like Height), this is certainly a line of inquiry to persue. We think that, however, doing this experiment robustly for many CVD traits (and with different methods) is certainly a distinctive paper in its own right. We have added a note in the Discussion on this point (L362-364).**

*24. Add number of participants from UKB in the summary statistics to estimate sample overlap (Sup Table 1). The analysis is presented as a two-sample MR, but as the authors note it is quite likely that the reliance on the UK Biobank leads to substantial sample overlap. Providing this information could help readers assess the degree of overlap.*

**Author Response: Based on comments from Reviewer #1, we have included this information in the supplement.**

*25. In the 1st paragraph of the discussion, the sentence "Our findings refute the notion that height, height-related nutritional status, or socioeconomic status meaningfully contributes to CAD risk" should be rephrased. In its current form, it reads as if socioeconomic status does not contribute to CAD risk. Moreover, the point estimate for the fully adjusted OR is 3.7% increase in the risk of CAD for a ~6.5cm decrease in height which could still be considered meaningful (especially for people that fall further away from the mean). I think this statement could be made more precise.*

**Author Response: We have adjusted this statement accordingly.**
