# Supplementary material for: Mendelian randomization analyses clarify the effects of height on cardiovascular diseases
Source: PLoS One. 2024 Jul 3;19(7):e0298786. doi: 10.1371/journal.pone.0298786 (PMC11221663; doi:10.1371/journal.pone.0298786)
Supplement: Attachment — Submitted filename: HuiEA_Height_CVD_MR_Rebuttal_r2_v1.docx [file pone.0298786.s025.docx]

**We sincerely thank the reviewers and editors for their additional reading of the manuscript and their criticisms and suggestions, and have minor revisions accordingly. Reviewer’s and Editor’s comments are in *italics*, and our specific responses are below in bold.**

**## Reviewer Comments**

*Review Comments to the Author*

***Reviewer #1:***

*I thank the authors for responding to my previous comments.

I have some minor suggestions, which are outlined below.

In their current format, the Figures are almost illegible. Please provide a better resolution.*

**Author Response: We think this has to do with the resolution downgrade for the .pdf packet that the reviewer’s receive. That said, we will absolutely make sure that high rez images are present in the final version.** *Abstract: "established risk factors" is a vague term. Could the authors be more precise and say instead cardiovascular, anthropometric, lung function, and lifestyle-related risk factors.*

**Author Response: Done.** *Line 77: “may complicate”: MVMR identifies confounding or mediating effects. I would suggest saying instead “confound or mediate”.*

**Author Response: Done.**

*Could the authors explain why proxies were not sought, especially in the multivariable MR were there were more missing SNPs?*

**Author Response: As the reviewer highlights, MVMR focused on an analysis of 1,906 SNPs (from our initial 2,037 SNP instrument), meaning that we lost about ~6.4% of data missing across all CVD traits we analyzed. Because analysis for that reduced instrument returned an OR and significance that matched the larger instrument, we opted not to pursue that here.**  *Line 128: “to the lung trait effect allele”. Do the authors mean that the alleles were harmonized to have an increasing effect on the lung trait?*

**Author Response: Yes, and we have revised to make this clear accordingly.** *Line 318: The authors here talk about other CV outcomes that the CAD, but start the paragraph with the statement on the CAD (which has been shown before). I suggest mentioning the results on ischemic stroke first (that’s the novelty of the analysis presented here), and then extend to the other tested CV outcomes, for which the results were non-significant.*

**Author Response: Appreciate the suggested framing and we have revised accordingly.** *Line 340: provides an independent and substantially: please correct grammar here (the word effect is missing).*

**Author Response: Done.**

*Point 22 in the rebuttal. I agree with Reviewer 2 that a two-step network MR as described by Burgess et al would be an asset in the manuscript, since the aim here is really to explore mediation. I strongly recommend adding this analysis.*

**Author Response: Following the suggest approach (**<https://academic.oup.com/ije/article/44/2/484/752297>)**, we perform this experiment using Height and FEV1 and FVC each. Using the Shrine et al data, we found that the ORs were virtually unchanged, indicating that the expected direct effect was basically identical to the UVMR estimate. We have added this result to the main text (at the end of the section “Genetically determined lung function measures is not causally associated with susceptibility to CAD”, L303-308).**
